# Behavioural cue reactivity to alcohol-related and non-alcohol-related stimuli among individuals with alcohol use disorder: An fMRI study with a visual task

**DOI:** 10.1371/journal.pone.0229187

**Published:** 2020-07-17

**Authors:** Shou Fukushima, Hironori Kuga, Naoya Oribe, Takeo Mutou, Takefumi Yuzuriha, Hiroki Ozawa, Takefumi Ueno

**Affiliations:** 1 Department of Neuropsychiatry, Nagasaki University, Nagasaki, Japan; 2 Department of Clinical Research, National Hospital Organization, Hizen Psychiatric Medical Center, Saga, Japan; 3 Department of Psychiatry, Michinoo Hospital, Nagasaki, Japan; 4 Department of Neuropsychiatry, Kyushu University, Fukuoka, Japan; Tokyo Metropolitan Institute of Medical Science, JAPAN

## Abstract

Patients with alcohol use disorder (AUD) have difficulty controlling their alcohol cravings and thus exhibit increased use and early relapse. Although patients tend to respond more strongly to alcohol-related images than to non-alcohol-related images, few researchers have examined the factors that modulate cravings. Here, we examined whole-brain blood oxygen level-dependent (BOLD) responses to behavioural cues in individuals with AUD and in healthy controls (HCs). The participants included 24 patients with AUD and 15 HCs. We presented visual cues consisting of four beverage-related images (juice, drinking juice, sake, and drinking sake), and the cue reactivity of AUD participants was contrasted with that of HC participants. Multiple comparisons revealed that the AUD group had lower BOLD responses than the HC group in the left precuneus (p = 0.036) and the left posterior cingulate cortex (PCC) (p = 0.044) to images of drinking juice and higher BOLD responses than the HC group in the left PCC (p = 0.044) to images of drinking sake. Furthermore, compared to the HCs, the AUD patients had decreased BOLD responses associated with cue reactivity to drinking juice in the left precuneus during the periods from 15 to 18 s (p = 0.004, df = 37) and 18 to 21 s (p = 0.002, df = 37). Our findings suggest that HCs and AUD patients differ in their responses not to images of alcoholic beverages but to images related to alcohol-drinking behaviour. Thus, these patients appear to have different patterns of brain activity. This information may aid clinicians in developing treatments for patients with AUD.

## Introduction

According to the World Health Organization (WHO), 3.3 million deaths every year result from the harmful use of alcohol, accounting for 5.9% of all deaths in the world [1]. The harmful use of alcohol is a causal factor in more than 200 diseases and injuries [2]. Overall, 5.1% of the global burden of disease and injury is attributable to alcohol, as measured in disability-adjusted life years [1]. Beyond health problems, the harmful use of alcohol is associated with significant social and economic losses for individuals and for society at large.

As the American Psychiatric Association (APA) guidelines suggest, not only abstinence from alcohol use but also reduction or moderation of alcohol use may be an appropriate initial goal of treatment for alcohol use disorder (AUD) from a harm-reduction perspective. However, craving induction in people with AUD can lead to compulsive drinking or reward anticipation. In research on substance-use relapse, exposure to substance-related cues (such as the sight or smell of alcoholic beverages) has been found to evoke elevations in subjective craving and physiological arousal and increase the likelihood of substance use [3]. Thus, craving appears to be an important predictor of negative outcomes such as increased use and early relapse in AUD patients [4]. However, some patients are not sensitive to craving before relapse. Furthermore, the mechanisms underlying craving remain unclear.

Previous studies investigating craving have suggested that the posterior cingulate cortex (PCC) plays a role in craving and relapse to alcohol use [5,6]. The PCC is a primary node of the default mode network (DMN) [7,8], which may be related to deficits in brain function in AUD patients. The PCC encodes relevant information from visual sensory systems to evaluate emotional content [9] and is involved in internally directed cognition, such as memory retrieval and planning [10]. This region plays a crucial role in integrating incoming memory with existing knowledge to create a coherent representation of the event [11]. While the PCC has been extensively studied in AUD patients through task-based studies on craving and AUD patients have been found to have greater responsiveness to alcohol-related images than images of non-alcoholic beverages [12], few studies have examined the factors that contribute to craving in AUD patients.

Although several studies have reported findings from human brain imaging, we are not aware of any research describing the relationship between drinking behaviour and cue reactivity among the many fMRI studies of AUD. AUD is classified as a substance use disorder by DSM-5 but is also a behavioural disorder. In AUD patients, we hypothesized that the presentation of stimuli involving alcohol-drinking behaviour may be accompanied by stronger brain activation than stimuli that simply contain alcoholic beverages. To address the question of whether and how the PCC and other brain regions related to the DMN are implicated in the deficits, we examined possible alterations in brain function in patients with AUD using functional MRI (fMRI) with beverage image cues. We anticipated that such alterations might be correlated with changes in cognitive function in the patient group.

## Materials and methods

### Participants

Demographic and clinical data are shown in Table 1. The sample consisted of 15 healthy controls (HCs), and 24 patients with AUD. Twenty-three participants were then diagnosed with severe AUD according to the DSM-5 criteria [13], and one participant was diagnosed with moderate AUD. We measured cognitive impairment using the Mini–Mental State Examination (MMSE). All participants were between 25 and 60 years of age. The HCs were screened using the Structured Clinical Interview (SCID)–non-patient edition [14], and the HCs were confirmed not to have any present or previous mental health problems.

**Table 1:** Demographic and clinical characteristics of the participant groups.

	AUD (N = 24)	HC (N = 15)	F or t	df	p-value
Age (mean, SD)	47.5 ± 8.6	46.7 ± 7.9	0.30	37	0.77
Sex (male), N (%)	17 (71)	10 (67)	0.27	37	0.79
Handedness^a^ (mean, SD)	87.7 ± 39.5	89.5 ± 14.9	1.80	37	0.87
Years of education (mean, SD)	12.8 ± 2.4	16.4 ± 2.9	0.00	37	< 0.001^★^
AUDIT score (mean, SD)	28.3 ± 7.1	1.7 ± 2.5	/	/	< 0.001
DSM-5 criteria met (mean, SD)	8.9 ± 2.1	0.20 ± 0.6	/	/	< 0.001
MMSE score (mean, SD)	28.5 ± 1.6	/			
Duration of illness (mean, SD)	8.7 ± 6.9	/			
Number of hospital admissions (mean, SD)	2.5 ± 1.4	/			
Neurologic symptoms^b^ (yes), N (%)	10 (42)	/			

Values are means ± SD unless otherwise indicated. HC, healthy control; AUD, alcohol use disorder; AUDIT, Alcohol Use Disorders Identification Test; MMSE, Mini–Mental State Examination.

^a^Based on the total score on the Annett Handedness Scale.

^b^History of withdrawal convulsions or hallucinations.

^★^Patients with AUD had significantly fewer years of education than HCs according to a t-test.

The participants with AUD were recruited from a pool of patients who had been hospitalized at our institution and who subsequently joined the alcohol rehabilitation programme (ARP) to recover from AUD. All AUD patients were screened using the Alcohol Use Disorders Identification Test (AUDIT [15]). We performed MRI in AUD participants 1–2 months after admission so that it would not be confounded by withdrawal convulsions or hallucinations.

### Design and ethical approval

**Fig 1:**
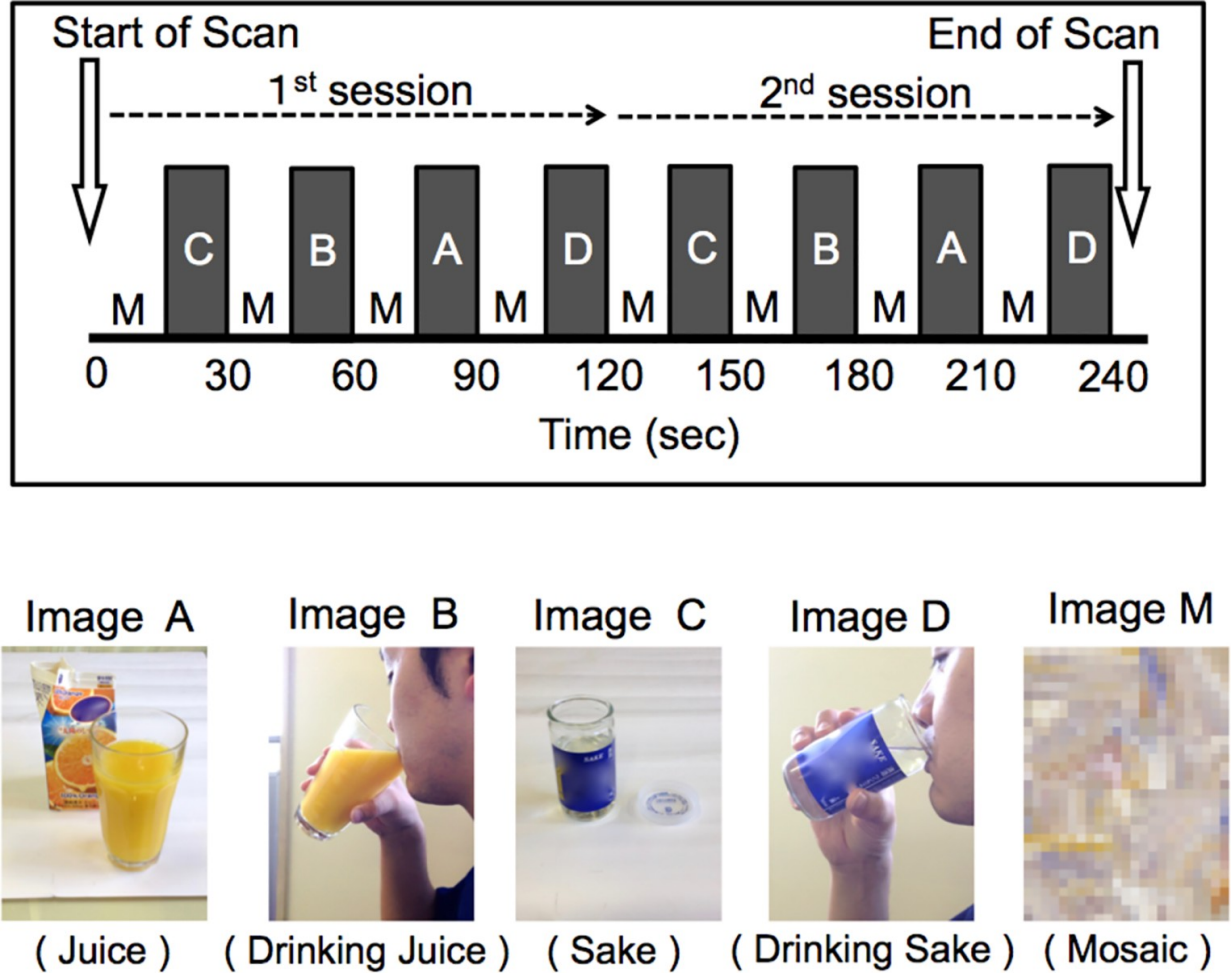
Study design and presenting images. We presented four beverage-related images (Image A, juice; Image B, drinking juice; Image C, sake; and Image D, drinking sake) and a mosaic image (Image M) to participants in a block design. Sake is rice wine. One session consisted of four rest blocks and four beverage blocks, and two sessions took a total of 240 s. Beverage images were presented in order of valence (for example, “C-B-A-D” in this figure), and each beverage image was presented twice in each fMRI session. In each MRI session, we presented the brand of each beverage in the images with permission from the copyright holder. Due to requests from the copyright holders of the juice and sake brands that we used, we obscured the beverage brands with mosaics in this report.

This study had a prospective, intervention-based cross-sectional design. The study intervention involved presenting images of beverages (alcoholic and non-alcoholic). The protocol and informed consent form were approved by the ethics committee of the National Hospital Organization Hizen Psychiatric Medical Center. After receiving a complete description of the study, all participants signed an informed consent form. The individual who is included in Fig 1 in this manuscript has given written informed consent (as outlined in PLOS consent form) to publish these case details.

### Stimuli and procedure

The methods were based on a previous study [16]. In the informed consent process, we explained to all participants that the tasks included the presentation of images, but we did not describe the content of the images. The participants completed eye tests, and the participants with poor vision were given glasses to wear during the MRI.

The participants were asked to lie in a supine position on a bed inside the MRI scanner while wearing headphones. Each participant’s head was restrained by padding behind the neck and between the head and the coil. Participants were asked to keep their head still inside the scanner and to focus on a fixation cross presented on a screen. All visual stimuli were presented on the screen.

We presented the visual stimuli using a 2-min block-design paradigm with eight blocks of 15 s of rest (viewing a mosaic image) and eight blocks of 15 s of stimulus presentation (viewing a beverage-related image). We presented four beverage-related images (Image A, juice; Image B, drinking juice; Image C, sake; and Image D, drinking sake) (Fig 1) in a pseudo-random order. We presented a mosaic image (Image M) (Fig 1) as a baseline image, and we obtained the contrast between T2* signals elicited by each picture (Image A, B, C, or D) and the mosaic image. This table was used to assign random test sequences to the participants. Sake is a type of rice wine, and patients with AUD in Japan often drink this type of alcohol because it has a high alcohol content (15% alcohol by volume) and is inexpensive (approximately 2.5 USD per bottle), portable, and convenient to drink; 19 of the 24 AUD participants in this study stated that sake was their preferred alcoholic beverage (Fig 1). To confirm that the participants had paid attention, we asked them after the MRI scan to list the pictures they had seen, and all participants named all five types of pictures in the correct sequence.

Each block used one image. The order of images was counterbalanced across participants, and each beverage image was presented twice. In total, we presented eight stimuli for two minutes in each fMRI session.

### Data acquisition

We conducted MRI using a 1.5-T Philips scanner with a standard head coil located at the National Hospital Organization Hizen Psychiatric Medical Center. We obtained functional images using the following standard sequence parameters: gradient-echo echo-planar imaging (EPI); repetition time (TR) = 3000 ms; echo time (TE) = 45 ms; flip angle = 90°; field of view (FOV) = 230 × 230 mm; matrix = 64 × 64; 60 axial slices with a slice thickness of 4 mm with no slice gap. We acquired a high-resolution T1-weighted 3D anatomical image for each participant between the functional data trials.

### Image processing

Raw image DICOM files were converted to the NIFTI format using MRI-Convert (Version 2.0, Lewis Center for Neuroimaging, Oregon). Image processing and statistical analyses were performed using the statistical parametric mapping software SPM12 [17] with MATLAB R2015a. The first five volumes of each EPI image run were excluded to allow the MR signal to reach a state of equilibrium. All volumes of the functional EPI images were realigned to the first volume of each session to correct for participant motion. These images were managed with slice-timing correction, and the mean functional EPI image was then spatially co-registered with the anatomical T1 images. Each co-registered T1-weighted anatomical image was normalized to a standard T1 template image (ICBM 152), which defined the Montreal Neurological Institute (MNI) space. The parameters from this normalization process were then applied to each functional image. The normalized functional images were smoothed with a 3D 8-mm full width at half-maximum (FWHM) Gaussian kernel. The time-series data at each voxel were temporally filtered using a high-pass filter with a cutoff of 128 s.

### Statistical analysis

We used one-way analyses of variance (ANOVAs), t-tests, and Wilcoxon rank-sum tests to assess group differences in the demographic variables. We performed fMRI statistical analysis on the preprocessed EPIs with the general linear model (GLM) using a two-level approach [18]. The model consisted of boxcar functions convolved with the canonical haemodynamic response function, which were then used as regressors in the regression analysis. Six head motion parameters, derived from realignment processing, were also used as regressors to reduce motion-related artefacts. On the first level of analysis, individual contrast images for each stimulus versus rest (the mosaic image) were computed and taken to the second level for random-effects inference. On the second level, contrast images for stimuli as the within-subject factors were submitted to two groups (AUD and HC) as the between-subject factors in a full-factorial ANOVA with education as a covariate. All fMRI results are reported at a significance level of p < 0.05, familywise error (FWE)-corrected (voxel-level corrected), or p < 0.05, FWE cluster-corrected across the whole brain with the initial voxel threshold at p < 0.001.

**Fig 2:**
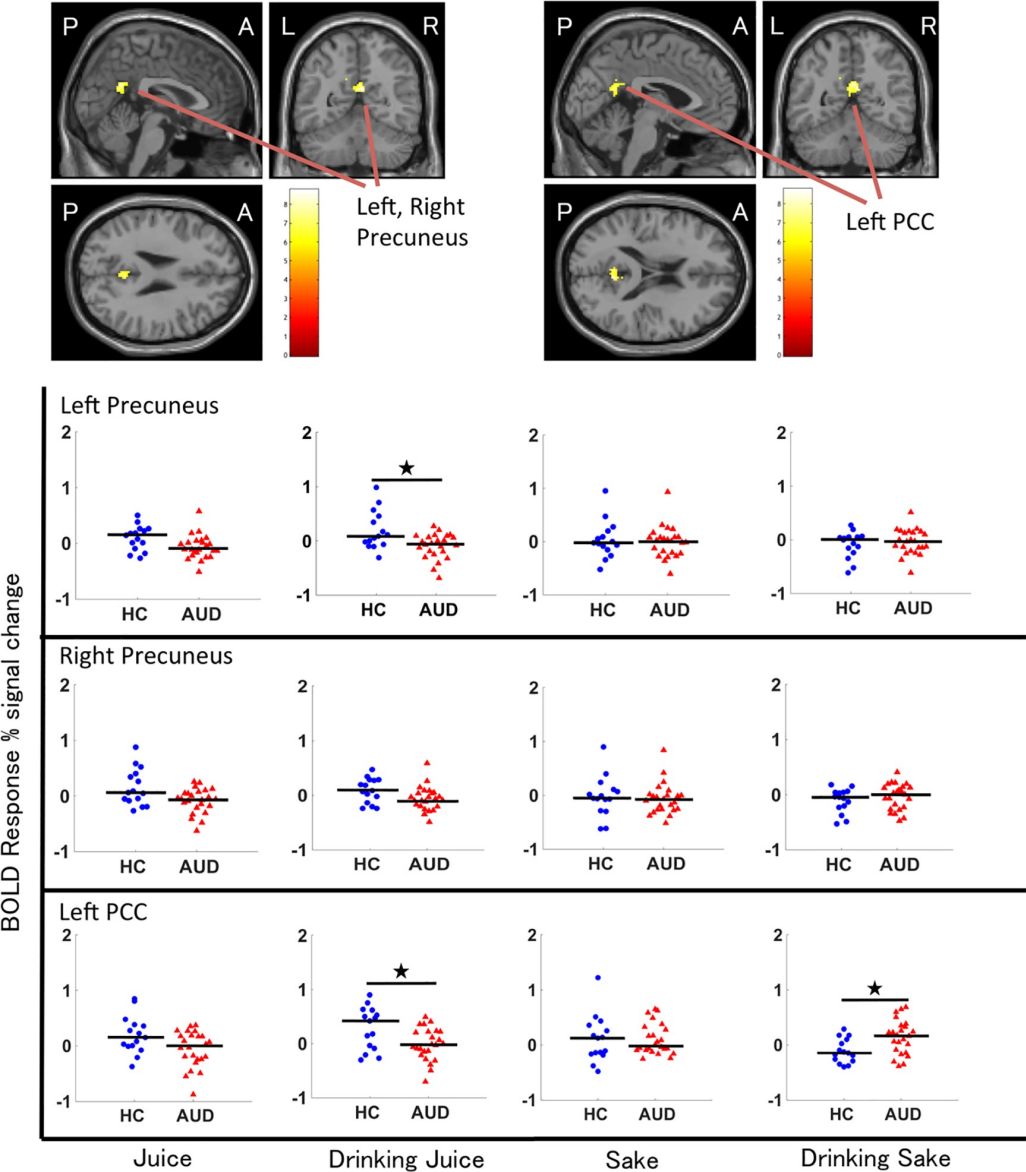
Regions where fMRI analyses revealed significant stimuli-by-group interactions. Coloured bars represent the F-values of the interactions (cluster-level FWE-corrected *p* < 0.05). The scatter diagram shows the BOLD responses to the four beverage images (juice, drinking juice, sake, and drinking sake) in healthy controls (marked “HCs”; n = 15, blue dots) and participants with AUD (marked “AUD”; n = 24, red dots) in the left precuneus, right precuneus, and left PCC. The bars show the median values. ^★^*p* < 0.05.

We extracted the contrast values in the regions of interest (ROIs) using MarsBar (http://marsbar.sourceforge.net). We chose three regions (the left precuneus, the right precuneus and the left PCC) because they contained significant stimulus-by-group interactions. For each stimulus, we compared the BOLD responses in the ROIs between the two groups (AUD and HC) via SPSS (ver. 24). To test the differences between the two groups, we used t-tests for data that were normally distributed and the Wilcoxon rank-sum test for data that were not normally distributed. We performed 11 sets of t-tests or Wilcoxon rank-sum tests to investigate the cue reactivity differences in brain regions between AUD patients and HCs under 12 conditions: three regions by four beverage pictures (Fig 2). We applied the false discovery rate (FDR) to the results of the t-tests or Wilcoxon rank-sum tests to examine multiple comparisons using SPSS according to Benjamini & Hochberg’s method. These comparison results are reported at a significance level of q < 0.05.

**Fig 3:**
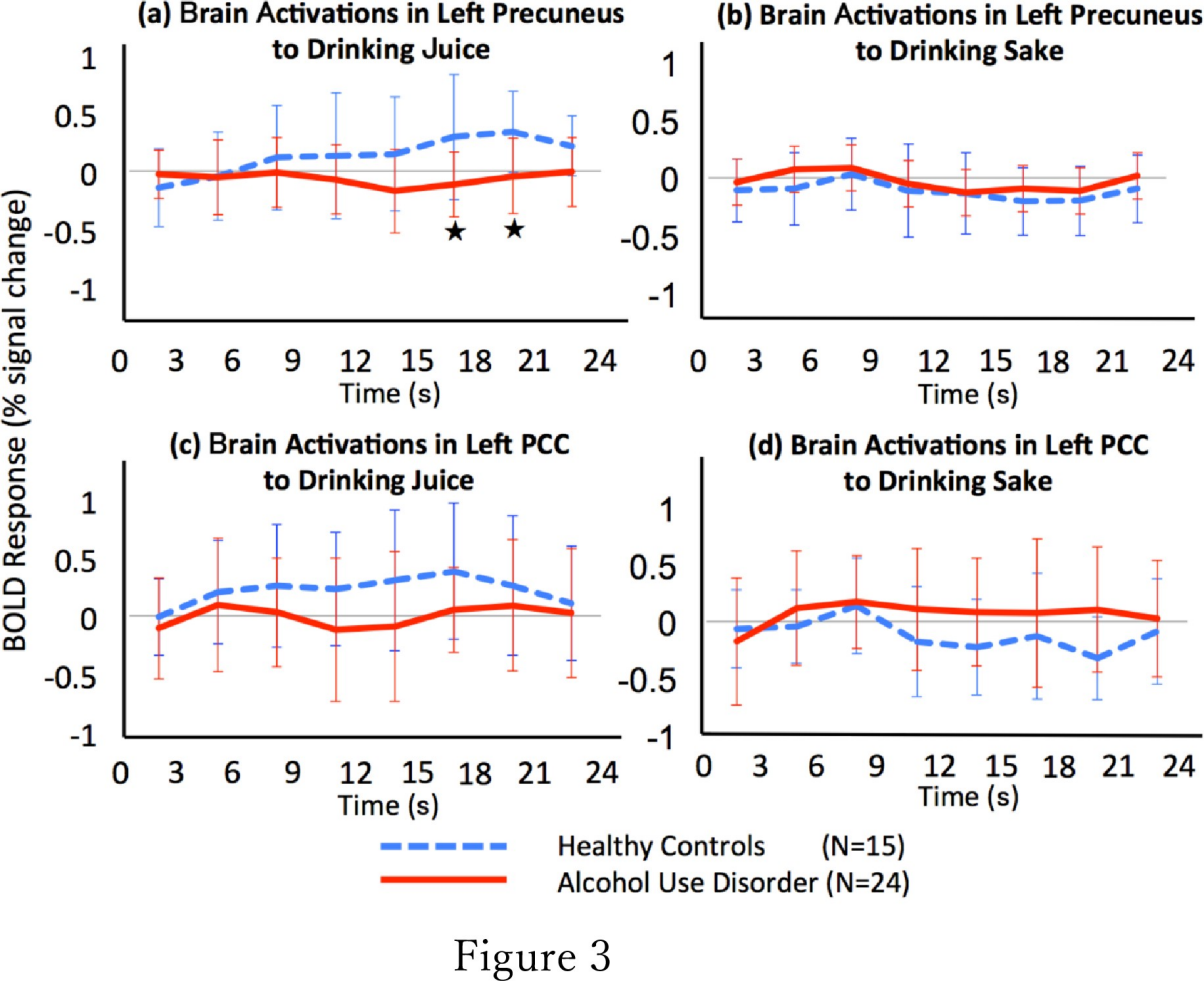
The time course of BOLD activation associated with behavioural cue reactivity for drinking juice or drinking sake in the left precuneus or left PCC. The x-axis indicates time (s), and the y-axis indicates percentage of BOLD signal change. The blue dotted lines and red lines indicate the percentages of BOLD signal change in the HC and AUD groups, respectively. The bars show each standard error. Compared with the HC group, the AUD group had a significantly decreased BOLD response in the left precuneus to the drinking juice stimulus from 15 to 21 s (^★^*p* < 0.0071) (a).

We used mixed-effect regression models in SPSS (ver. 24) with random intercepts to test our hypothesis that the time course of brain activity with respect to the behavioural cue reactivity of drinking juice would differ between the AUD and HC groups, with education as a covariate. We chose the contrast values with respect to the cue of drinking juice in the left precuneus, because we found a significant time-by-group interaction in that region. We performed seven sets of t-tests to investigate the cue reactivity differences in brain regions between AUD patients and HCs under seven conditions: 3 s -6 s, 6 s-9 s, 9 s-12 s, 12 s-15 s, 15 s-18 s, 18 s-21 s, 21 s-24 s (Fig 3). We applied the Bonferroni corrected to the results of the t-tests to examine multiple comparisons.

In the AUD participants, we calculated Spearman correlations in SPSS (ver. 24) to assess between-group differences in the correlations between brain activation elicited by beverage images and demographic measurements (age, sex, handedness, and education) or clinical measurements (duration of illness; number of hospital admissions; past history of neurologic symptoms; preferred alcoholic beverage; and doses of antipsychotics, acamprosate, and antidepressant drugs). In the HC participants, we calculated the Spearman correlations in SPSS (ver. 24) to assess between-group differences in the correlations between brain activation and demographic measurements.

### Outcome

The primary outcome was the difference in the BOLD responses to four beverage images (images of beverages or drinking beverages) between participants with AUD and HCs. The BOLD response was measured using the statistical parametric mapping software SPM12 (Wellcome Department of Cognitive Neurology, London, United Kingdom) with MATLAB R2015a (The Math Works Inc., Natick, MA).

## Results

### Group demographic data

Means, SDs, and frequencies for clinical and demographic variables are presented in Table 1. We found no significant group differences in the demographic data with respect to age, sex, and handedness. However, the participants with AUD had significantly lower levels of education than the HCs (p < 0.001, F = 0.0, df = 37), as well as higher AUDIT scores and numbers of DSM-5 AUD criteria met (p < 0.001, F = 14.398, df = 37). Seven patients were medicated with antipsychotics (only atypical antipsychotics; chlorpromazine equivalents = 180.5 ± 132.3 mg), and 17 patients were unmedicated. Within the patient group, three participants received acamprosate (1443 ± 508.7 mg), and six received antidepressant drugs. Ten patients had a history of neurologic symptoms, such as withdrawal convulsions or hallucinations. There were no differences in the number of hospital admissions, comorbid conditions, or preferred alcohol type.

### Main effects of group and stimulus and stimulus-by-group interaction

As shown in Table 2 and Fig 2, repeated-measures ANOVAs showed several significant group differences. The main effect of group was associated with significant differences in activity in the right precentral cortex (10, -26, 62; cluster size = 2,416; F[11,28] = 25.32; cluster-level, FWE-corrected p < 0.001), the left precentral cortex (-2, -26, 66; cluster size = 2,416; F[11,28] = 14.06; cluster-level, FWE-corrected p < 0.001; -4, -18, 58; cluster size = 712; F[11,28] = 25.32; cluster-level, FWE-corrected p < 0.001), the left supramarginal cortex (-60, -40, 24; cluster size = 2,416; F[11,28] = 15.74; cluster-level, FWE-corrected p = 0.025), the left cerebellum exterior (-8, -36, -22; cluster size = 1,008; F[11,28] = 15.05; cluster-level, FWE-corrected p < 0.001), the left hippocampus (-22, -30, -10; cluster size = 1,008; F[11,28] = 14.44; cluster-level, FWE-corrected p < 0.001), and the exterior right cerebellum (4, -36, -22; cluster size = 1,008; F[11,28] = 13.30; cluster-level, FWE-corrected p < 0.001). In addition, the main effect of stimulus was associated with significant differences in activity in the middle temporal gyrus (52, -64, 6; cluster size = 9,136; F[5,71] = 28.66; cluster-level, FWE-corrected p < 0.001; peak-level, FWE-corrected p < 0.001), in the right inferior occipital gyrus (46, -64, 0; cluster size = 9,136; F[5,71] = 28.66; cluster-level, FWE-corrected p < 0.001; peak-level, FWE-corrected p < 0.001), in the right angular gyrus (42, -56, 14; cluster size = 9,136; F[5,71] = 28.66; cluster-level, FWE-corrected p < 0.001; peak-level, FWE-corrected p = 0.083), in the right occipital pole (14, -92, 18; cluster size = 4,272; F[5,71] = 28.66; cluster-level, FWE-corrected p < 0.001; peak-level, FWE-corrected p = 0.002), in the left occipital pole (-8, -96, 12; cluster size = 4,272; F[5,71] = 28.66; cluster-level, FWE-corrected p < 0.001; peak-level, FWE-corrected p = 0.054), in the right lingual gyrus (6, -76, -2; cluster size = 4,272; F[5,71] = 28.66; cluster-level, FWE-corrected p = 0.021; peak-level, FWE-corrected p = 0.017) in the left inferior occipital gyrus (-22, -90, 0; cluster size = 616; F[5,71] = 28.66; cluster-level, FWE-corrected p = 0.021; peak-level, FWE-corrected p = 0.746), and in the left occipital fusiform gyrus (-18, -90, -8; cluster size = 616; F[5,71] = 28.66; cluster-level, FWE-corrected p = 0.021; peak-level, FWE-corrected p = 1.000). Furthermore, we found a significant stimulus-by-group interaction in the left precuneus, the right precuneus, and the left PCC (cluster-level uncorrected p = 0.002).

**Table 2:** fMRI results for anatomical regions, seed voxel coordinates (MNI), and F-values for significant stimulus-by-group interactions.

Cluster size (mm^3^)	MNI coordinates			K_E_	F-value	Anatomical region
	x	y	z			
832	-2	-54	18	115	5.71	Left precuneus
	6	-54	20			Right precuneus
	0	-48	24			Left posterior cingulate cortex

BOLD, blood oxygen level-dependent; MNI, Montreal Neurological Institute.

The results are thresholded at a cluster-corrected p < 0.05.

### BOLD contrast for behavioural cue reactivity associated with drinking juice vs. drinking sake in the precuneus and PCC

To determine the direction of the stimulus-by-group interaction, we extracted contrast values by identifying the PCC and precuneus as ROIs. A post hoc test corrected for multiple comparisons revealed the following significant group differences for Image B (drinking juice) and Image D (drinking sake); HCs > AUD in terms of BOLD responses to Image B in the left precuneus (p = 0.003, t = -3.12) and left PCC (p = 0.011, t = -2.67), and AUD > HCs in terms of BOLD responses to Image D in the left PCC (p = 0.010, t = 2.73). In the right precuneus, however, our test showed no significant differences in BOLD responses between the participants with AUD and HCs. Furthermore, in the three ROIs, our tests showed no significant differences in BOLD responses to Image A (juice) and Image C (sake) between the AUD participants and HCs. Fig 2 shows the regions where the fMRI analyses revealed significant stimulus-by-group interactions and a scatter diagram. There was a group difference between the distribution trends.

In the left PCC, the median of the BOLD responses to Image B in the AUD participants was lower than that in HCs, but the median of the BOLD responses to Image D in the AUD participants was higher than that in the HCs. In the left precuneus, however, this inverse relationship was absent.

### The time course of BOLD activation associated with behavioural cue reactivity for drinking juice vs. drinking sake

We found significant group differences in the time course of BOLD responses with a linear mixed-effect model and a post hoc t-test (F = 2.726, p = 0.010). Specifically, we found that, compared to the HC group, the AUD group exhibited a decrease in cue reactivity in the left precuneus during the periods from 15 s to 18 s (p = 0.004, t = -3.044, df = 37) and from 18 s to 21 s (p = 0.002, t = -3.392, df = 37) after the onset of Image B (Fig 3A). Conversely, we found no significant difference between the two groups in the time course for cue reactivity associated with Image D in the left precuneus, Image B in the left PCC, or Image D in the left PCC (Fig 3B–3D).

### Correlations between brain activation and demographic/clinical measurements

In the AUD participants, we found no statistically significant between-group differences in the correlations between brain activation elicited by beverage-related images and demographic measurements (age, sex, handedness, and education) or clinical measurements (duration of illness; number of hospital admissions; history of neurologic symptoms; preferred alcoholic beverage; and doses of antipsychotics, acamprosate, and antidepressant drugs). In the HC participants, we also found no statistically significant between-group differences in the correlations between brain activation and demographic measurements.

## Discussion

We collected fMRI data for admitted AUD patients and HCs while they viewed substance-related and behaviour-related visual cues of alcohol and non-alcoholic beverages. To the best of our knowledge, this is the first study to demonstrate biological differences in brain function associated with strong visual behavioural cues regarding alcoholic and non-alcoholic beverages in AUD patients. As we expected, compared to the HCs, the AUD patient group had increased activation in the PCC to the drinking alcohol stimulus. This observation suggests that patients with AUD had higher responses to the stimulus associated with alcohol-drinking behaviour than to a stimulus that simply contained an image of an alcoholic beverage.

In our study, the patients with AUD had reduced behavioural cue reactivity–associated BOLD activation to the non-alcoholic beverages in the left PCC and the left precuneus. A recent meta-analysis of fMRI studies using alcohol cue reactivity demonstrated BOLD activation in several brain regions in AUD patients [19,20], including the PCC and precuneus. This observation suggests that PCC plays an important role in addiction and relapse [21]. When the PCC is activated by a visual stimulus, information from episodic memory is extracted and integrated with existing knowledge. The human precuneus is associated with several basic cognitive activities in the resting-state condition. These functions include the collection and evaluation of information, self-referential mental activity, extraction of episodic memory, emotion, and anxiety [22]. Previous studies have found that AUD patients have impairments in the PCC and precuneus. Our study suggests that in patients with AUD, the functional changes in the PCC may affect the circuits involved in episodic memory.

In this study, the patients with AUD displayed stronger reactivity to drinking behavioural cues, but not simple beverage cues, than HCs. According to previous research, both theory and empirical evidence suggest that attention bias for alcohol cues is associated with symptoms of AUD [23]. Furthermore, patients with AUD may experience stronger episodic memory than HCs upon seeing an alcohol-drinking stimulus, considering the function of the PCC and precuneus.

In the left precuneus, we found that the time course of brain activity in the AUD and HC groups significantly differed with respect to the behavioural cue reactivity to drinking juice, especially during the period from 15 s to 18 s and from 18 s to 21 s after the onset of the juice-drinking stimulus presentation. These results indicated that in the left precuneus, the patients with AUD experienced deactivation in the latter half of the juice-drinking stimulus trials. Additionally, these data suggested that the patients exhibited decreased responses in the precuneus when they viewed an unfavourable stimulus, leading to a delayed response. The precuneus is associated with the evaluation of information and emotion. Compared with the HC group, the AUD group had lower behavioural cue reactivity–associated BOLD responses to the non-alcohol images. Thus, the precuneus of the patients with AUD may have only weakly evaluated the juice-drinking stimulus.

In interpreting the current study, it is important to consider several possible limitations. First, as most of the patients we recruited were severe AUD patients (23 severe and one moderate according to the DSM-5 criteria), selection bias should be considered. Mild and moderate AUD patients should be examined using the same approach to determine whether the BOLD activity that we observed was a state-dependent endophenotype. Second, given that the sample of participants was small, the statistical power for assessing brain activity was limited. Future studies with larger numbers of participants are needed to determine whether brain BOLD activity can differentiate BOLD patterns elicited by the behavioural cue reactivity towards alcohol and non-alcohol beverages in AUD patients. Third, there might be a possibility that we could not completely exclude the effect of the image-specific stimulus from the alcohol-specific stimulus because we used only one sake image for the feasibility of the experience. But we could not use several stimulus images, because we needed to keep the MRI scans time shortly. Fourth, the choice of ROIs for the fMRI could be considered arbitrary. The regions were selected on the basis of their involvement in specific cue-elicited BOLD responses. The choice of ROIs could have been broadened to include other regions known to be involved in reward circuitry associated with addiction (e.g., the ACC, ventral striatum, and amygdala). Despite these limitations, this line of research may contribute to the understanding of the neural circuitry underlying addiction, which may have important implications for our understanding of alcohol addiction pathology and treatment.

## Conclusions

Our study showed that, compared with controls, patients with AUD exhibited signs of functional brain impairment. Taking a harm-reduction approach, the APA guidelines and the Japanese domestic guidelines suggest that not only abstinence from alcohol use but also reduction or moderation of alcohol use may be an appropriate initial goal for AUD treatment.

Clinically, however, individuals with severe AUD generally have great difficulty moderating their alcohol use. When such individuals drink alcoholic beverages, they often exacerbate their physical, mental, domestic, and/or social problems because of their difficulties in controlling their alcohol use. We believe that it is very difficult for those patients to remain safe in their daily lives while pursuing the treatment goal of reducing or moderating their alcohol intake.

The difficulty that AUD patients face in moderating their alcohol intake may be related to the neural differences indicated by our study. Our results may help to explain the challenges encountered in such moderation efforts.

## References

[1] World Health Organization. Global status report on alcohol and health. Geneva, Switzerland: World Health Organization; 2014.

[2] World Health Organization International statistical classification of mental and behavioural disorders. Geneva, Switzerland: World Health Organization; 1992.

[3] Carter BL, Tiffany ST. Meta-analysis of cue-reactivity in addiction research. Addiction. 1999;94: 327–340. 10605857

[4] Seo D, Lacadie CM, Tuit K, Hong KI, Constable RT, Sinha R. Disrupted ventromedial prefrontal function, alcohol craving, and subsequent relapse risk. JAMA Psychiatry. 2013;70: 727–739. 10.1001/jamapsychiatry.2013.762 23636842 PMC3788824

[5] Zakiniaeiz Y, Scheinost D, Seo D, Sinha R, Constable RT. Cingulate cortex functional connectivity predicts future relapse in alcohol dependent individuals. Neuroimage Clin. 2017;13: 181–187. 10.1016/j.nicl.2016.10.019 27981033 PMC5144743

[6] Chase HW, Eickhoff SB, Laird AR, Hogarth L. The neural basis of drug stimulus processing and craving: An activation likelihood estimation meta-analysis. Biol Psychiatry. 2011;70: 785–793. 10.1016/j.biopsych.2011.05.025 21757184 PMC4827617

[7] Brewer JA, Worhunsky PD, Gray JR, Tang YY, Weber J, Kober H. Meditation experience is associated with differences in default mode network activity and connectivity. Proc Natl Acad Sci U S A. 2011;108: 20254–20259. 10.1073/pnas.1112029108 22114193 PMC3250176

[8] Raichle ME, MacLeod AM, Snyder AZ, Powers WJ, Gusnard DA, Shulman GL. A default mode of brain function. Proc Natl Acad Sci U S A. 2001;98: 676–682. 10.1073/pnas.98.2.676 11209064 PMC14647

[9] Vogt BA, Vogt L, Laureys S. Cytology and functionally correlated circuits of human posterior cingulate areas. Neuroimage. 2006;29: 452–466. 10.1016/j.neuroimage.2005.07.048 16140550 PMC2649771

[10] Leech R, Kamourieh S, Beckmann CF, Sharp DJ. Fractionating the default mode network: Distinct contributions of the ventral and dorsal posterior cingulate cortex to cognitive control. J Neurosci. 2011;31: 3217–3224. 10.1523/JNEUROSCI.5626-10.2011 21368033 PMC6623935

[11] Bird CM, Keidel JL, Ing LP, Horner AJ, Burgess N. Consolidation of complex events via reinstatement in posterior cingulate cortex. J Neurosci. 2015;35: 14426–14434. 10.1523/JNEUROSCI.1774-15.2015 26511235 PMC4623223

[12] Myrick H, Anton RF, Li X, Henderson S, Drobes D, Voronin K, et al Differential brain activity in alcoholics and social drinkers to alcohol cues: Relationship to craving. Neuropsychopharmacology. 2004;29: 393–402. 10.1038/sj.npp.1300295 14679386

[13] American Psychiatric Association. Diagnostic and statistical manual of mental disorders. Washington DC: American Psychiatric Association; 2013.

[14] First MB, Spitzer RL, Gibbon M, Williams J. Structure clinical interview for DSM-IV-TR axis I disorders-non-patient edition (SCID-I/NP, 11/2002 revision). New York, NY: Biometrics Research, New York State Psychiatric Institute; 2002.

[15] Saunders JB, Aasland OG, Babor TF, de la Fuente JR, Grant M. Development of the alcohol use disorders identification test (AUDIT): WHO collaborative project on early detection of persons with harmful alcohol consumption—II. Addiction. 1993;88: 791–804. 10.1111/j.1360-0443.1993.tb02093.x 8329970

[16] Kuga H, Onitsuka T, Hirano Y, Nakamura I, Oribe N, Mizuhara H, et al Increased BOLD signals elicited by high gamma auditory stimulation of the left auditory cortex in acute state schizophrenia. EBioMedicine. 2016;12: 143–149. 10.1016/j.ebiom.2016.09.008 27649638 PMC5672078

[17] SPM 12 (2014) Wellcome Department of Imaging Neuroscience. London, UK, http://www.fil.ion.ucl.ac.uk/spm.

[18] Friston KJ, Holmes AP, Worsley KJ, Poline JP, Frith CD, Frackowiak RSJ. Statistical parametric maps in functional imaging: A general linear approach. Hum Brain Map. 1994;2: 189–210.

[19] Noori HR, Cosa Linan A, Spanagel R. Largely overlapping neuronal substrates of reactivity to drug, gambling, food and sexual cues: A comprehensive meta-analysis. Eur Neuropsychopharmacol. 2016;26: 1419–1430. 10.1016/j.euroneuro.2016.06.013 27397863

[20] Schacht JP, Anton RF, Myrick H. Functional neuroimaging studies of alcohol cue reactivity: A quantitative meta-analysis and systematic review. Addict Biol. 2013;18: 121–133. 10.1111/j.1369-1600.2012.00464.x 22574861 PMC3419322

[21] Krienke UJ, Nikesch F, Spiegelhalder K, Hennig J, Olbrich HM, Langosch JM. Impact of alcohol-related video sequences on functional MRI in abstinent alcoholics. Eur Addict Res. 2014;20: 33–40. 10.1159/000349909 23921439

[22] Luo X, Guo L, Dai XJ, Wang Q, Zhu W, Miao X, et al Abnormal intrinsic functional hubs in alcohol dependence: Evidence from a voxelwise degree centrality analysis. Neuropsychiatr Dis Treat. 2017;13: 2011–2020. 10.2147/NDT.S142742 28814870 PMC5546828

[23] Field M, Cox WM. Attentional bias in addictive behaviors: a review of its development, causes, and consequences. Drug and Alcohol Dependence. 2008;97:1–20. 10.1016/j.drugalcdep.2008.03.030 18479844

