# Correction: Behavioural cue reactivity to alcohol-related and non-alcohol-related stimuli among individuals with alcohol use disorder: An fMRI study with a visual task

**DOI:** 10.1371/journal.pone.0242531

**Published:** 2020-11-12

**Authors:** Shou Fukushima, Hironori Kuga, Naoya Oribe, Takeo Mutou, Takefumi Yuzuriha, Hiroki Ozawa, Takefumi Ueno

There is an error in affiliation 1 for authors Shou Fukushima and Hiroki Ozawa. The correct affiliation 1 is: Department of Neuropsychiatry, Nagasaki University Graduate School of Biomedical Sciences, Nagasaki, Japan
